# Association between monocyte-to-lymphocyte ratio and cardiovascular diseases: insights from NHANES data

**DOI:** 10.1186/s13098-025-01640-9

**Published:** 2025-03-24

**Authors:** Xiaowan Li, Liyan Zhang, Yingying Du, Yiru Shen, Yuanzhi Gong, Junjie Wang, Juan Zhou, Sheng Wang

**Affiliations:** 1 Intensive Care Medical Center, Tongji Hospital, School of Medicine, Tongji University, Shanghai, 200065 China; 2 Department of Critical Care Medicine, Shanghai Tenth People’s Hospital, School of Medicine, Tongji University, Shanghai, 200072 China

**Keywords:** Monocyte-to-lymphocyte ratio, Cardiovascular disease, Population-based study, Cross-sectional study, NHANES

## Abstract

**Background:**

This study intends to examine any possible correlation between monocyte-to-lymphocyte ratio (MLR) and cardiovascular diseases (CVD).

**Methods:**

Data from the 1999–2020 National Health and Nutrition Examination Survey (NHANES) in the USA were analyzed. Heart attacks, angina pectoris, congestive heart failure (CHF), coronary heart disease (CHD), and stroke were all covered by CVD. The independent relationships between these cardiovascular events and MLR levels, as well as other inflammatory indices (system inflammation response index (SIRI), aggregate index of systemic inflammation (AISI), and C-reactive protein-to-albumin ratio (CAR)), were investigated. Furthermore, interaction tests and subgroup analysis were performed. Diagnostic capacities were also predicted and compared using receiver operating characteristic (ROC) curves.

**Results:**

Males made up 49.63% of the 46,289 people who were recruited in this study. The prevalence of CVD and its events were as follows: CHF at 2.99%, CHD at 3.72%, angina pectoris at 2.57%, heart attacks at 3.94%, and stroke at 3.48%, with CVD itself at 7.98%. MLR and CVD were positively correlated. Specifically, smooth curve fittings also found a non-linear relationship between MLR and CVD. Moreover, higher MLR levels were linked to increased rates of CHF, CHD, and strokes. SIRI wa*s* also found to have a positive correlation with CVD. MLR outperformed other inflammatory indices (SIRI, AISI, and CAR) in terms of discriminative capacity and accuracy in predicting CVD, CHF, CHD, angina pectoris, heart attack, and stroke, according to ROC analysis.

**Conclusions:**

Compared with other inflammatory indicators (SIRI, AISI, and CAR), MLR appears to be a better inflammatory index for predicting CVD, CHF, CHD, angina pectoris, heart attack, and stroke. American adults with elevated MLR and SIRI should be aware of the possible harm caused by CVD. Causal inference is, however, limited by the cross-sectional design and dependence on self-reported data. Further longitudinal studies are needed to validate these findings.

**Supplementary Information:**

The online version contains supplementary material available at 10.1186/s13098-025-01640-9.

## Introduction

Cardiovascular diseases (CVD) continue to be a major global cause of death, endangering healthy lifespans and driving up healthcare expenses, making it a major public health concern [1]. CVD has been gradually rising in both prevalence and fatality rates. While mortality from CVD increased from 12.1 million in 1990 to 18.6 million in 2019, the number of people with CVD increased from 271 million in 1990 to 523 million in 2019 [2]. Considering these increasing rates, it is critical to look into the causes of CVD and to find preventative measures meant to postpone and lessen its prevalence. Known risk factors for CVD include diabetes, hypertension, smoking, inflammation, and dyslipidemia [3–5]. Hypertension and dyslipidemia also can exacerbate inflammatory responses, while smoking and diabetes further amplify systemic inflammation, thereby indirectly affecting the CVD [6, 7]. Consequently, inflammation has become a major modifiable risk factor that is essential for creating clinically effective treatment plans that stop CVD from starting and progressing.

Numerous investigations have examined the noteworthy correlation between inflammation and CVD [3, 8–14]. Previous research found positive associations between aggregate index of systemic inflammation (AISI) and heart attack and stroke [8–10]. System inflammation response index (SIRI) has been significantly linked to illnesses such as myocardial infarction, stroke, coronary heart disease (CHD), and congestive heart failure (CHF) [15–18]. However monocyte-to-lymphocyte ratio (MLR) is getting more and more attention, which integrates monocyte count (MC) and lymphocyte count (LC). Consequently, elevated MLR may be a sign of compromised immune responses and enhanced inflammatory responses [19]. This dual role makes MLR a potentially more comprehensive biomarker compared to other inflammatory markers. A substantial association between higher MLR levels and a higher prevalence of CHF was also documented by Zhang et al. [16]. MLR is a reliable and efficient marker for CHD, according to ten-year nationwide research [15]. Recent studies highlight that the MLR may serve as a novel biomarker for cardiovascular risk in individuals with type 2 diabetes (T2D), where chronic inflammation and oxidative stress exacerbate cardiovascular outcomes [20, 21]. However, the relationship between MLR and CVD within the broader population remains unexplored. To our knowledge, this is the first population-based study to comprehensively compare MLR with other inflammatory indices (SIRI, AISI, CAR) in predicting multiple CVD events, while accounting for non-linear relationships and subgroup variations.

## Methods

### Data sources

Cross-sectional data was supplied by NHANES. The National Center for Health Statistics (NCHS) administers NHANES surveys to assess the physical and nutritional health of the US population that is not institutionalized [22]. While the NHANES survey data is still in its two-year repetition cycle, it is being updated. Among the enrolled participants, the NHANES study design’s stratified multi-stage probabilistic method yields a comparatively high representation. The ethical review committee of the NCHS has approved all survey methods and protocols used in NHANES. Please go to the official NHANES website to learn more about the preparation and implementation (http://www.cdc.gov/nchs/nhanes).

### Study population

**Fig. 1:**
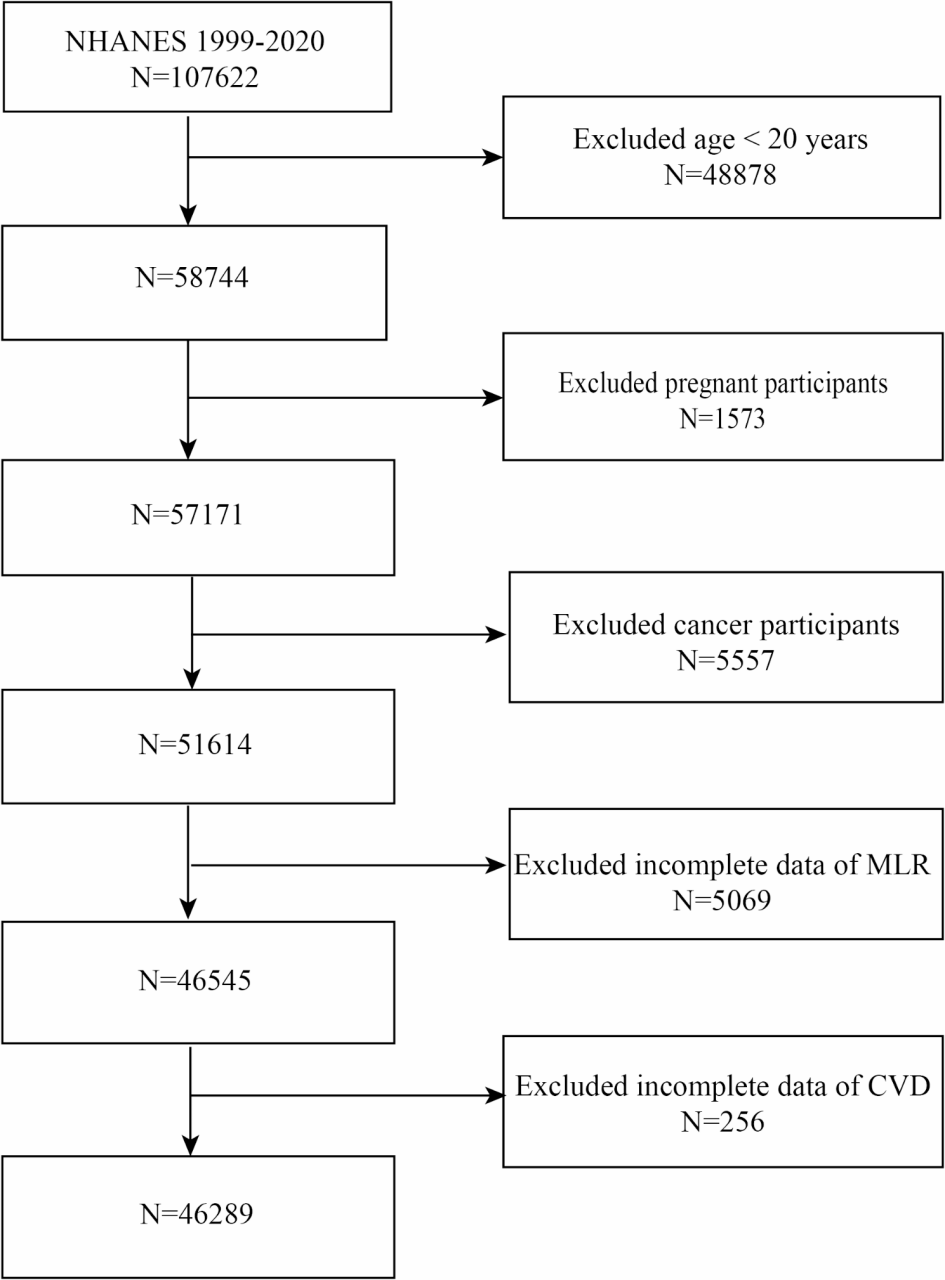
Flowchart of the sample selection from NHANES 1999–2020

We analyzed data from participants enrolled in the NHANES surveys conducted between 1999 and 2020. Participants with cancer or pregnancy were excluded because these conditions can significantly alter inflammatory markers and confound the association between MLR and CVD [23, 24]. Following the removal of patients with cancer (*n* = 5,557), pregnancy (*n* = 1,573), missing MLR (*n* = 5,069), CVD (*n* = 256), and age < 20 years (*n* = 48,878) from the study (*n* = 107,622), 46,289 eligible participants were enrolled (Fig. 1).

### Definition of MLR and CVD

The Beckman Coulter technique for counting and sizing is employed in the procedures to determine complete blood count (CBC) parameters, in addition to automatic equipment for mixing and diluting samples. Using the NHANES Standard Biochemistry Profile, the albumin (ALB) value was determined. The albumin concentration is measured using the dye bromocresol purple (BCP). Blood was drawn in the morning following a fast. Information on albumin, C-reactive protein (CRP), MC, platelet count (PC), neutrophil count (NC), and LC was gathered. The subsequent formulas were applied for the computation of the indices: MLR = MC/LC, SIRI = NC × MC/LC, AISI = NC × PC × MC/LC, and C-reactive protein-to-albumin ratio (CAR) = CRP/ALB. NHANES’ stringent quality control procedures, which include standardized laboratory protocols, the use of validated equipment, and routine calibration checks, guarantee the accuracy of these measurements.

The definition of CVD was derived from the survey questionnaire answers provided by the study participants. CVD was considered if a participant reported experiencing angina pectoris, heart attack, CHD, CHF, or stroke [25]. Consequently, any affirmative answer to these specific criteria was interpreted as indicating the presence of CVD. While self-reported data may have limitations, NHANES employs rigorous quality control measures, including standardized interview protocols and cross-validation with medical records when available, to ensure data accuracy [22].

### Selection of covariates

In our analysis, various demographic variables were controlled for, including sex, age, race, marital status, family income to poverty ratio (PIR), and education level. In addition to these, we incorporated several laboratory and anthropometric variables as covariates, such as total bilirubin, serum total calcium, smoking status, alcohol consumption, body mass index (BMI), total cholesterol (TC), high-density lipoprotein-cholesterol (HDL-C), aspartate aminotransferase (AST), and alanine aminotransferase (ALT).

As confounders in our analysis, we also included health status disparities such as diabetes, hypertension, diabetic kidney disease (DKD), chronic obstructive pulmonary disease (COPD), Rheumatoid arthritis (RA), asthma, and close relative had heart attack. There are three components to the definition of hypertension employed in this study. Initially, hypertension is identified through self-reporting, as indicated by the questionnaire item “Ever told you had hypertension.” The second criterion pertains to the measurement of average systolic blood pressure (SBP) or diastolic blood pressure (DBP) exceeding 130 or 80 mmHg, respectively [26]. The third criterion for identifying participants with hypertension is based on the use of antihypertensive medications, as captured by the survey item regarding “taking hypertension prescription.” The definition used for diabetes also included three components. The first component was self-reported diabetes, while the second was about insulin or diabetes medication use. Finally, individuals with diabetes were identified using clinical criteria, specifically fasting plasma glucose (FPG) ≥ 7.0 mmol/L and hemoglobin A1c (HbA1c) levels or using glycated hemoglobin greater than 6.5%. Low estimated glomerular filtration rate (eGFR) (less than 60 mL/min/1.73 m^2^) or albuminuria (urinary albumin-to-creatinine ratio (UACR) ≥ 30 mg/g) in diabetic patients were used to diagnose DKD [27].

According to earlier studies, self-reported physician diagnoses for COPD, RA, asthma, and close relative had heart attack were defined [28–30]. It was confirmed using a composite of three self-reported COPD questionnaire items: “Has a doctor ever told you that you have chronic bronchitis?”, “Has a doctor ever told you that you have emphysema?”, and “Has a doctor or other health professional ever told you that you have COPD?”. The COPD group was made up of individuals who selected “yes” for any one of the three questions. RA asked the following questions: “Has a doctor or other health professional ever told you that you had arthritis?”. The response options were “yes” or “no”. When a participant selected “yes,” they were asked to respond to the following question: “Which type of arthritis was it?“. The participants were asked whether they had ever been diagnosed with asthma by a doctor or other health professional to diagnosis asthma. Finally, the participants were asked whether close relative had heart attack.

### Statistical analysis

All statistical analyses adhered to the recommendations provided by the Centers for Disease Control (CDC), accounting for the complex multi-stage cluster survey design and employing the appropriate NHANES sampling weights. Continuous variables were summarized using standard error (SE), while categorical variables were represented as percentages. Participants were categorized based on MLR tertiles, and differences among these groups were evaluated using either a weighted t-test or a chi-square test. To investigate the relationship between MLR and CVD, three distinct multivariable logistic regression models were tested. Model 1 was unadjusted for covariates. In Model 2, adjustments were made for age, sex, and race. Sex, age, race, marital status, education level, BMI, smoking status, alcohol consumption, TC, HDL-C, AST, ALT, PIR, total bilirubin, serum total calcium, RA, DKD, asthma, close relative had heart attack, COPD, hypertension and diabetes were all considered while adjusting for these factors in Model 3. To deal with non-linear relationships, generalized additive models (GAM) and smooth curve fitting were used. In order to examine threshold effects, we fitted a two-segment linear regression model (segmented regression model) to each interval and used the log-likelihood ratio test to compare the results to the one-line model (non-segmented). In order to identify breakpoints, we employed a two-step recursive technique [31]. The relationship between them was assessed through subgroup analyses using stratified multivariable logistic regression models. Subgroup analyses were performed by stratifying participants into these categories: age (< 40, 41–60, > 60 years), sex (male, female), BMI (< 25, 25–30, ≥ 30 kg/m²), diabetes (yes, no), and hypertension (yes, no). These subgroups were chosen to explore potential effect modification by key demographic and clinical factors. Additionally, we assessed the predictive capabilities of MLR and other inflammatory markers (SIRI, AISI, and CAR) for CVD, CHF, CHD, angina pectoris, heart attack, and stroke using receiver operating characteristic (ROC) curves and comparing the area under the curve (AUC) values. To evaluate the relative importance of inflammatory markers in predicting cardiovascular events, we applied the Standardized Domination Statistic (SDS) method. This approach quantifies the contribution of each predictor within a model by calculating its dominance score, allowing for a comparison of their relative importance in predicting outcomes. While median imputation was utilized for continuous variables, mode imputation was employed for categorical variables to deal with missing values. While this approach minimizes bias, it assumes that data are missing at random and does not account for the uncertainty associated with missing data, potentially underestimating variability and leading to biased standard errors. We conducted our statistical analyses using R version 4.1.3 and the Empower software package. The threshold for statistical significance was set at a two-tailed *p*-value less than 0.05.

## Results

### Participants’ characteristics at baseline

This study included 46,289 participants with an average age of 48.66 ± 17.54 years old. Males made up 49.63%, while females made up 50.37%. The prevalence rates of CVD, CHF, CHD, angina pectoris, heart attack, and stroke were found to be 7.98%, 2.99%, 3.72%, 2.57%, 3.94%, and 3.48%, respectively. CVD, CHF, CHD, angina pectoris, heart attack, and stroke were all more common in those with the higher MLR tertile (Table 1) (all *p* < 0.05).

**Table 1:** Baseline characteristics according to MLR tertiles

MLR	Overall	Tertile 1	Tertile 2	Tertile 3	*P*-value
		(0.03–0.22)	(0.22–0.30)	(0.30–2.50)	
N	46,289	14,952	15,825	15,512	
MLR	0.28 ± 0.12	0.17 ± 0.03	0.25 ± 0.02	0.40 ± 0.12	< 0.001
SIRI	1.21 ± 0.88	0.68 ± 0.31	1.06 ± 0.44	1.88 ± 1.12	< 0.001
AISI	311.93 ± 285.45	181.44 ± 110.29	275.74 ± 231.79	474.55 ± 363.83	< 0.001
CAR	0.11 ± 0.23	0.10 ± 0.17	0.09 ± 0.16	0.13 ± 0.33	< 0.001
Age, years					< 0.001
20–40	17,058 (36.81%)	6349 (42.42%)	6180 (39.03%)	4529 (29.15%)	
41–60	15,952 (34.43%)	5572 (37.23%)	5556 (35.09%)	4824 (31.05%)	
> 60	13,327 (28.76%)	3046 (20.35%)	4099 (25.89%)	6182 (39.79%)	
Sex, n (%)					< 0.001
Male	22,995 (49.63%)	5609 (37.48%)	7888 (49.81%)	9498 (61.14%)	
Female	23,342 (50.37%)	58 (62.52%)	7947 (50.19%)	6037 (38.86%)	
Race, n (%)					< 0.001
Mexican American	8328 (17.97%)	3008 (20.10%)	3031 (19.14%)	2289 (14.73%)	
Other Hispanic	4116 (8.88%)	1428 (9.54%)	1445 (9.13%)	1243 (8.00%)	
Non-Hispanic White	19,173 (41.38%)	4486 (29.97%)	6603 (41.70%)	8084 (52.04%)	
Non-Hispanic Black	10,128 (21.86%)	4224 (28.22%)	3160 (19.96%)	2744 (17.66%)	
Other Races	4592 (9.91%)	1821 (12.17%)	1596 (10.08%)	1175 (7.56%)	
Education level, n (%)					< 0.001
Less than high school	12,441 (26.85%)	4185 (27.96%)	4207 (26.57%)	4049 (26.06%)	
High school or GED	10,776 (23.26%)	3343 (22.34%)	3628 (22.91%)	3805 (24.49%)	
Above high school	23,061 (49.77%)	7427 (49.63%)	7983 (50.41%)	7651 (49.25%)	
Others	58 (0.13%)	11 (0.07%)	17 (0.11%)	30 (0.19%)	
Marital status, n (%)					< 0.001
Married	20,373 (52.54%)	6526 (51.74%)	7118 (53.70%)	6729 (52.13%)	
Never married	7147 (18.43%)	2398 (19.01%)	2461 (18.57%)	2288 (17.73%)	
Living with a partner	2908 (7.50%)	1091 (8.65%)	1000 (7.54%)	817 (6.33%)	
Others	8347 (21.53%)	2598 (20.60%)	2676 (20.19%)	3073 (23.81%)	
BMI, n (%)					< 0.001
Normal weight	13,481 (29.58%)	4206 (28.43%)	4573 (29.30%)	4702 (30.98%)	
Overweight	15,284 (33.53%)	4710 (31.84%)	5339 (34.21%)	5235 (34.49%)	
Obese	16,813 (36.89%)	5877 (39.73%)	5695 (36.49%)	5241 (34.53%)	
Smoking status, n (%)					< 0.001
≥100 cigarettes lifetime	20,758 (44.84%)	6228 (41.63%)	6944 (43.87%)	7586 (48.91%)	
< 100 cigarettes lifetime	25,539 (55.16%)	8731 (58.37%)	8884 (56.13%)	7924 (51.09%)	
PIR, n (%)					< 0.001
Low income	12,984 (30.83%)	3975 (29.29%)	4559 (31.72%)	4450 (31.41%)	
Medium income	15,897 (37.75%)	5077 (37.41%)	5377 (37.42%)	5443 (38.42%)	
High income	13,229 (31.42%)	4521 (33.31%)	4435 (30.86%)	4273 (30.16%)	
Alcohol consumption, n (%)					< 0.001
yes	28,782 (78.40%)	9087 (79.02%)	9985 (79.61%)	9710 (76.64%)	
no	7928 (21.60%)	2412 (20.98%)	2557 (20.39%)	2959 (23.36%)	
Hypertension, n (%)	24,394 (52.64%)	7257 (48.49%)	7977 (50.38%)	9160 (58.96%)	< 0.001
Diabetes, n (%)	7378 (15.92%)	2355 (15.73%)	2321 (14.66%)	2702 (17.39%)	< 0.001
RA	1032 (2.24%)	311 (2.08%)	300 (1.90%)	421 (2.73%)	< 0.001
DKD	2885 (6.26%)	752 (5.04%)	859 (5.45%)	1274 (8.28%)	< 0.001
Asthma	6101 (13.25%)	2021 (13.56%)	2032 (12.89%)	2048 (13.32%)	0.217
Close relative had heart attack	4157 (12.31%)	1378 (12.26%)	1357 (11.81%)	1422 (12.89%)	0.048
COPD	257 (2.74%)	51 (1.80%)	62 (1.93%)	144 (4.32%)	< 0.001
TC, mg/dL	194.36 ± 41.44	197.78 ± 42.36	195.15 ± 40.20	190.27 ± 41.44	< 0.001
ALT, U/L	25.40 ± 24.67	24.55 ± 16.97	25.56 ± 20.28	26.05 ± 33.40	< 0.001
AST, U/L	25.26 ± 19.07	24.35 ± 13.48	25.02 ± 15.33	26.38 ± 25.83	0.004
HDL-C, mg/dL	52.57 ± 15.86	52.33 ± 15.44	52.40 ± 15.77	52.99 ± 16.34	0.042
Total bilirubin, mg/dL	0.65 ± 0.32	0.62 ± 0.29	0.65 ± 0.31	0.68 ± 0.35	< 0.001
Serum total calcium, mg/dL	9.41 ± 0.38	9.41 ± 0.38	9.41 ± 0.37	9.40 ± 0.38	0.054
CHF, n (%)	1385 (2.99%)	247 (1.65%)	312 (1.97%)	826 (5.33%)	< 0.001
CHD, n (%)	1722 (3.72%)	288 (1.93%)	442 (2.79%)	992 (6.41%)	< 0.001
Angina pectoris, n (%)	1190 (2.57%)	252 (1.69%)	350 (2.21%)	588 (3.80%)	< 0.001
Heart attack, n (%)	1823 (3.94%)	342 (2.29%)	460 (2.91%)	1021 (6.58%)	< 0.001
Stroke, n (%)	1611 (3.48%)	411 (2.75%)	444 (2.81%)	756 (4.87%)	< 0.001
CVD, n (%)	3696 (7.98%)	998 (6.67%)	1279 (8.08%)	2425 (15.61%)	< 0.001

MLR, monocyte-to-lymphocyte ratio; SIRI, systemic inflammation response index; AISI, aggregate index of systemic inflammation; CAR, C-reactive protein and albumin ratios; GED, general educational development; BMI, body mass index; PIR, family income to poverty ratio; RA, rheumatoid arthritis; DKD, diabetic kidney disease; COPD, chronic obstructive pulmonary disease; TC, total cholesterol; ALT, alanine aminotransferase; AST, aspartate aminotransferase; HDL-C, high-density lipoprotein-cholesterol; CHF, congestive heart failure; CHD, coronary heart disease; CVD, cardiovascular diseases

### Association between MLR and CVD

Table 2 shows how MLR and CVD are related. In Model 3, each unit increment in MLR and SIRI is associated with a 2.78-fold and 14% increase in the prevalence of CVD. A statistically significant correlation was maintained even after these inflammatory markers were changed to tertiles. Individuals in the highest tertile of MLR and SIRI exhibited a significantly elevated prevalence of CVD compared to those in the lowest tertile (*p* for trend < 0.05).

**Table 2:** Association between MLR and other inflammatory biomarkers with CVD

Index	Continuous or categories	Model 1^3^		Model 2^4^		Model 3^5^	
		OR^1^ (95%CI^2^)	*P*- value	OR (95%CI)	*P*- value	OR (95%CI)	*P*- value
MLR	MLR as continuous variable	25.49 (20.67, 31.44)	< 0.0001	3.74 (2.96, 4.73)	< 0.0001	3.78 (1.83, 7.82)	0.0003
	Tertile 1	Reference		Reference		Reference	
	Tertile 2	1.23 (1.13, 1.34)	< 0.0001	1.00 (0.91, 1.10)	0.9947	1.15 (0.87, 1.53)	0.3330
	Tertile 3	2.59 (2.40, 2.80)	< 0.0001	1.40 (1.28, 1.52)	< 0.0001	1.39 (1.05, 1.83)	0.0194
	*P* for trend	< 0.0001		< 0.0001		0.0143	
SIRI	SIRI as continuous variable	1.42 (1.38, 1.46)	< 0.0001	1.24 (1.20, 1.28)	< 0.0001	1.14 (1.05, 1.25)	0.0034
	Tertile 1	Reference		Reference		Reference	
	Tertile 2	1.34 (1.23, 1.45)	< 0.0001	1.22 (1.11, 1.33)	< 0.0001	1.04 (0.78, 1.37)	0.8086
	Tertile 3	2.47 (2.29, 2.67)	< 0.0001	1.78 (1.63, 1.94)	< 0.0001	1.34 (1.03, 1.75)	0.0322
	*P* for trend	< 0.0001		< 0.0001		0.0119	
AISI	AISI as continuous variable	1.01 (1.00, 1.01)	< 0.0001	1.01 (1.01, 1.02)	< 0.0001	1.01 (0.99, 1.02)	0.1718
	Tertile 1	Reference		Reference		Reference	
	Tertile 2	1.18 (1.09, 1.28)	< 0.0001	1.15 (1.05, 1.25)	0.0017	1.10 (0.85, 1.42)	0.4599
	Tertile 3	1.69 (1.57, 1.82)	< 0.0001	1.49 (1.38, 1.62)	< 0.0001	1.08 (0.84, 1.39)	0.5309
	*P* for trend	< 0.0001		< 0.0001		0.6177	
CAR	CAR as continuous variable	1.87 (1.64, 2.13)	< 0.0001	1.63 (1.41, 1.89)	< 0.0001	1.37 (0.99, 1.87)	0.0520
	Tertile 1	Reference		Reference		Reference	
	Tertile 2	1.58 (1.41, 1.76)	< 0.0001	1.22 (1.08, 1.37)	0.0017	1.06 (0.86, 1.31)	0.5774
	Tertile 3	2.13 (1.92, 2.37)	< 0.0001	1.49 (1.38, 1.62)	< 0.0001	1.16 (0.94, 1.44)	0.1615
	*P* for trend	< 0.0001		< 0.0001		0.1527	

In sensitivity analysis, MLR, SIRI, AISI, and CAR were converted from continuous variables to categorical variables (tertiles)

^1^OR: Odd ratio

^2^95% CI: 95% confidence interval

^3^Model 1: No covariates were adjusted

^4^Model 2: Adjusted for age, sex, and race

^5^Model 3: Adjusted for sex, age, race, marital status, education level, BMI, smoking status, alcohol consumption, TC, HDL-C, AST, ALT, PIR, total bilirubin, serum total calcium, RA, DKD, asthma, close relative had heart attack, COPD, hypertension and diabetes

**Fig. 2:**
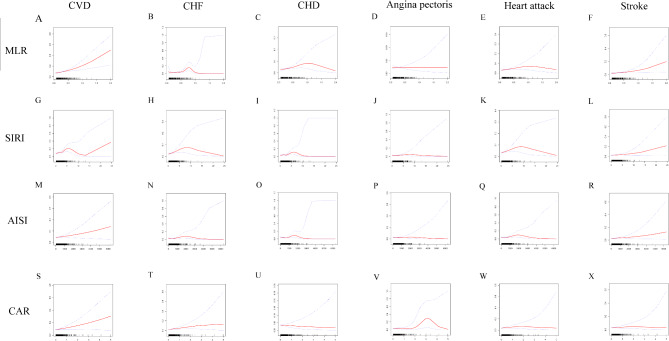
Smooth curve fitting for MLR and other inflammatory markers with CVD. (**A**) MLR and CVD; (**B**) MLR and CHF; (**C**) MLR and CHD; (**D**) MLR and angina pectoris; (**E**) MLR and heart attack; (**F**) MLR and stroke; (**G**) SIRI and CVD; (**H**) SIRI and CHF; (**I**) SIRI and CHD; (**J**) SIRI and angina pectoris; (**K**) SIRI and heart attack; (**L**) SIRI and stroke; (**M**) AISI and CVD; (**N**) AISI and CHF; (**O**) AISI and CHD; (**P**) AISI and angina pectoris; (**Q**) AISI and heart attack; (**R**) AISI and stroke; (**S**) CAR and CVD; (**T**) CAR and CHF; (**U**) CAR and CHD; (**V**) CAR and angina pectoris; (**W**) CAR and heart attack; (**X**) CAR and stroke

MLR and CVD were found to have non-linear connections with corresponding breakpoints of 0.16 according to GAM and smooth curve fittings (Fig. 2). The prevalence of CVD is positively correlated with MLR when it surpasses 0.16. To the left of the breakpoint, there was no significant association (Table 3).

**Table 3:** Threshold effect analysis of MLR and other inflammatory biomarkers on CVD using a two-piecewise linear regression model in model 3

	MLR	SIRI	AISI	CAR
	OR1(95%CI^2^) *P-* value	OR(95%CI)*P-* value	OR(95%CI)*P-* value	OR(95%CI) *P-* value
**CVD**				
Fitting by standard linear model	3.78 (1.83, 7.82) 0.0003	1.14 (1.05, 1.25) 0.0034	1.01 (0.99, 1.02) 0.1718	1.37 (0.99, 1.87) 0.0520
Fitting by two-piecewise linear model				
Breakpoint (K)	0.16	1.53	111.3	0.01
OR1 (< K )	0.01 (0.01, 20.90) 0.5257	1.33 (0.99, 1.78) 0.0567	1.00 (0.99, 1.01) 0.4126	0.75 (0.01, 1.27) 0.2308
OR2 (> K )	4.02 (1.91, 8.43) 0.0002	1.10 (0.98, 1.23) 0.1007	1.00 (0.99, 1.01) 0.1283	1.34 (0.98, 1.85) 0.0674
OR2 / OR1	0.67 (0.09, 1.42) 0.4174	0.83 (0.58, 1.17) 0.2881	1.00 (0.99, 1.01) 0.3838	0.02 (0.01, 1.55) 0.2341
Logarithmic likelihood ratio test *P*-value	0.032	0.288	0.390	0.222

Adjusted for sex, age, race, marital status, education level, BMI, smoking status, alcohol consumption, TC, HDL-C, AST, ALT, PIR, total bilirubin, serum total calcium, RA, DKD, asthma, close relative had heart attack, COPD, hypertension and diabetes

^1^OR: Odd ratio

^2^95% CI: 95% confidence interval

### Association between MLR and CVD events

We found that each unit increase in MLR is associated with a rise in the prevalence of CVD events, including CHF, CHD, and stroke, by 5.71, 2.12, and 2.39 times, respectively, after adjusting for all variables (Supplementary Table S1). A statistically significant correlation was maintained even when MLR was changed to tertiles. Individuals with the greatest MLR tertiles were more likely to experience CHF and CHD than those with the lowest tertile (*p* for trend < 0.05).

Additionally, we identified a non-linear relationship with threshold effects between SIRI and CAR in relation to the prevalence of CHF, with breakpoints determined at 2.83 and 0.02, respectively, after full adjustment (Supplementary Table S2).

### Subgroup analysis

**Fig. 3:**
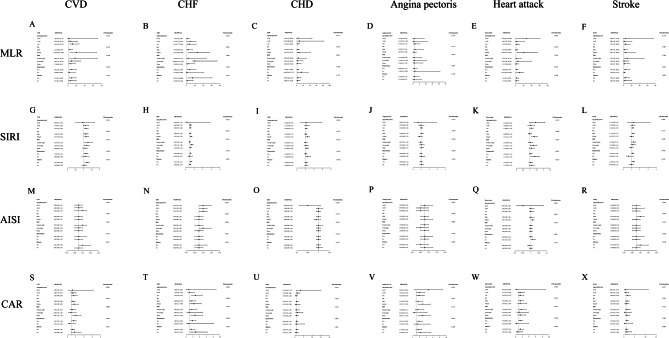
Subgroup analysis for the associations of MLR and other inflammatory markers with CVD. (**A**) MLR and CVD; (**B**) MLR and CHF; (**C**) MLR and CHD; (**D**) MLR and angina pectoris; (**E**) MLR and heart attack; (**F**) MLR and stroke; (**G**) SIRI and CVD; (**H**) SIRI and CHF; (**I**) SIRI and CHD; (**J**) SIRI and angina pectoris; (**K**) SIRI and heart attack; (**L**) SIRI and stroke; (**M**) AISI and CVD; (**N**) AISI and CHF; (**O**) AISI and CHD; (**P**) AISI and angina pectoris; (**Q**) AISI and heart attack; (**R**) AISI and stroke; (**S**) CAR and CVD; (**T**) CAR and CHF; (**U**) CAR and CHD; (**V**) CAR and angina pectoris; (**W**) CAR and heart attack; (**X**) CAR and stroke

Subgroup analysis indicated that age, sex, BMI, diabetes, and hypertension had no substantial impact on the relationship between MLR, SIRI, and CAR with CVD (*p* for interaction > 0.05) (Fig. 3). The subgroup analysis results also indicated a consistent link between CHD and angina pectoris with MLR across all groups. MLR and heart attack had a gender-dependent connection, with women being especially affected.

We further investigated the interaction effects of continuous variables (BMI and SBP) with MLR, SIRI, AISI, and CAR in predicting the prevalence of CVD (Supplementary Table S3). The results indicated that BMI and SBP, as continuous variables, significantly influenced the association between MLR and CVD (*p* < 0.05).

### ROC analysis

**Fig. 4:**
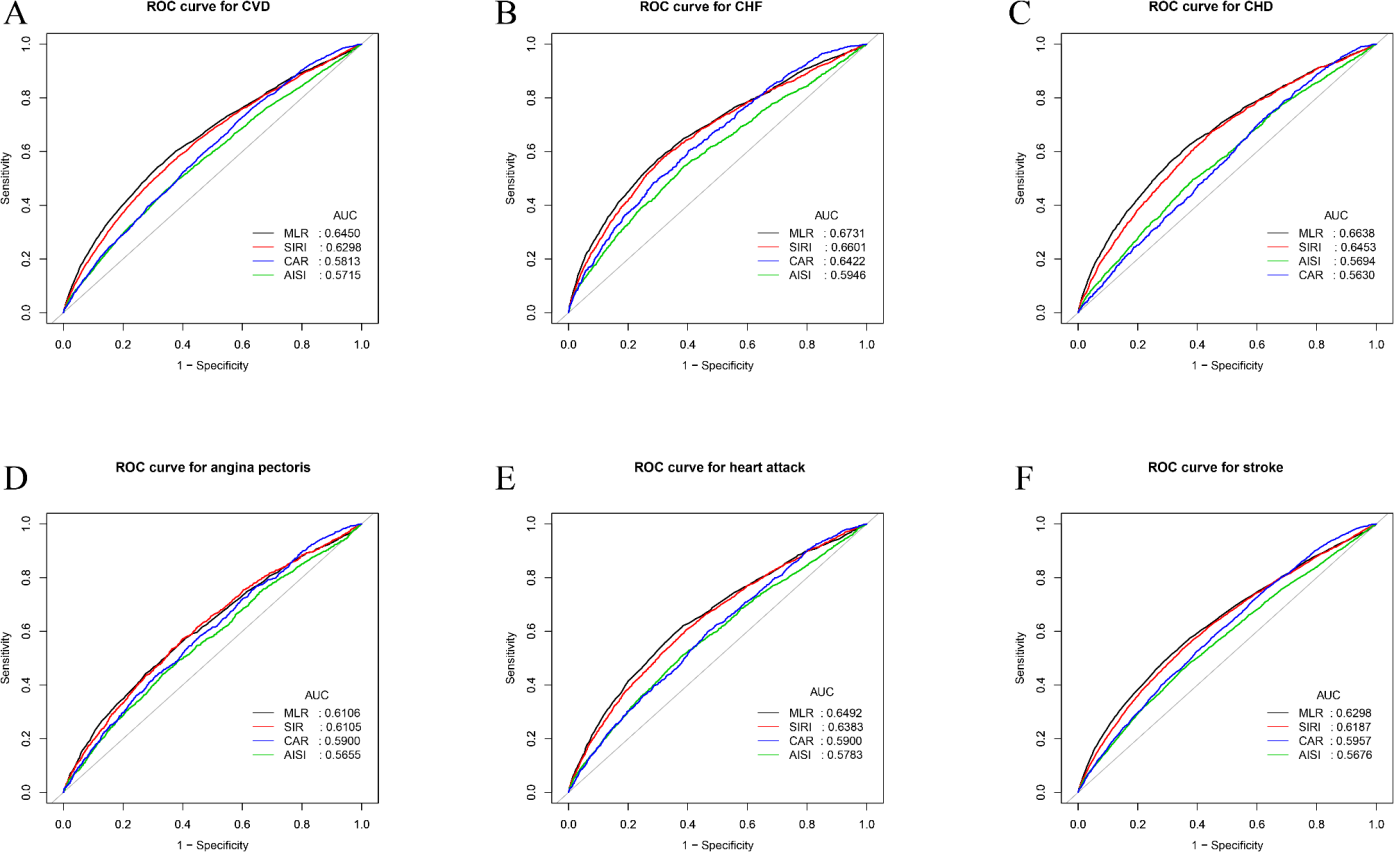
ROC curves and the AUC values of the four inflammatory markers (MLR, SIRI, AISI, and CAR) in diagnosing CVD, CHF, CHD, angina pectoris, heart attack, and stroke. (**A**) Four inflammatory markers were assessed to identify CVD; (**B**) Four inflammatory markers were assessed to identify CHF; (**C**) Four inflammatory markers were assessed to identify CHD; (**D**) Four inflammatory markers were assessed to identify angina pectoris; (**E**) Four inflammatory markers were assessed to identify heart attack; (**F**) Four inflammatory markers were assessed to identify stroke

For cardiovascular events, we computed the AUC to assess MLR’s prognostic accuracy in relation to other inflammatory markers (SIRI, AISI, and CAR) (Fig. 4). Our analysis revealed that MLR exhibited a superior AUC compared to the other inflammatory markers in predicting CVD, CHF, CHD, angina pectoris, heart attack, and stroke. Additionally, all differences in AUC values of the inflammatory indicators were statistically significant in comparison to MLR (all *p* < 0.05) (Supplementary Table S4). To evaluate the relative importance of these inflammatory markers in predicting cardiovascular events, we also employed the SDS method (Supplementary Table S5). The results demonstrated that MLR consistently exhibited the highest relative importance across CVD, CHF, CHD, angina pectoris, heart attack, and stroke, outperforming other markers (SIRI, AISI, CAR). These findings show that, in comparison to other inflammatory markers, MLR has a higher discriminative power and accuracy in predicting cardiovascular events.

## Discussion

We discovered that MLR and CVD were positively correlated in this cross-sectional investigation, which included 46,289 adults. The two were also shown to have a non-linear connection, with a breakpoint found at 0.16. Additionally, we observed a positive correlation between MLR and strokes, CHD, and CHF. ROC analysis and SDS method demonstrated that MLR may serve as the greater predictor for CVD, CHF, CHD, angina pectoris, heart attack, and stroke compared to other inflammatory markers (SIRI, AISI, and CAR). Further studies are warranted to validate these results and explore the potential clinical utility of MLR in risk stratification.

The connection between MLR and CVD in the general population are being examined for the first time in this investigation. The association between MLR and several cardiovascular disorders has been investigated in the past. CHF prevalence and MLR levels were revealed to be significantly positively correlated in cross-sectional research with 26,021 people [16]. A nationwide study analyzing 24,924 U.S. adults from NHANES found that increased SIRI, MLR, and LPR were strongly associated with the prevalence of CHD [15]. These results were supported by our investigation, which showed that the prevalence of CHF and CHD increased 5.71 and 2.12 times, respectively, for every unit rise in MLR levels. Additionally, our findings are consistent with previous research highlighting MLR as a predictive marker for strokes [32].

MLR has also been linked to a considerable risk of CVD in patients receiving hemodialysis (HD), peritoneal dialysis (PD), and chronic kidney disease (CKD), according to earlier research [33–36]. We are the first to show that the prevalence of CVD increases 2.78 times for every unit increase in MLR levels in the general population of the United States. A non-linear connection between MLR and CVD was also found, with a breakpoint at 0.16. A 3.02-fold increase in CVD prevalence was linked to every unit rise in MLR when it was above 0.16, but no meaningful association was seen to the left of this breakpoint. Awareness needs to be raised that high MLR is a marker for increased CVD risk, particularly among individuals with elevated inflammatory profiles. There may be underlying mechanisms behind this connection. One important factor in the development of CVD has been identified as chronic low-grade inflammation [37]. While lymphocytes, which are made up of T cells, B cells, and natural killer cells, represent the immune system’s regulatory mechanisms, monocytes are implicated in the inflammatory response. Therefore, higher MLR could be a sign of both weakened immune responses and enhanced inflammatory responses [19]. Our findings are consistent with previous studies highlighting MLR as a predictive marker for CVD in diverse populations [33–36]. However, differences in study design and population characteristics may explain variations in effect sizes.

The predictive value of MLR for the severity of coronary artery disease, cardiovascular mortality risk in patients with CKD, and the superiority of MLR in determining the severity of stable angina have also been investigated in previous studies [19, 38, 39]. Our research also suggests that MLR may be a more effective inflammatory marker for identifying CVD, CHF, CHD, angina pectoris, heart attacks, and stroke than SIRI, AISI, and CAR. This conclusion could be due to the ability of MLR to integrate the relative changes of monocytes and lymphocytes and reflect the human body’s status in chronic inflammation and immune responses. Additionally, MLR exhibits a robust resistance to external interferences and relatively good stability under pathological conditions [40, 41]. Given its ease of use and affordability, MLR could help identify subgroups of patients at high CVD risk, although further longitudinal studies are needed to confirm these findings.

Earlier research found one of the biggest risk factors for CVD is becoming older [42–44]. Important processes behind cardiovascular aging include age-related low-grade inflammation, oxidative stress, decreased nitric oxide (NO) bioavailability, mitochondrial dysfunction, decreased bioenergetic efficiency, elevated apoptosis, age-related autophagy decline, cellular senescence, and renin-angiotensin-aldosterone system activation [45, 46]. Our subgroup analysis and interaction tests support this view, demonstrating that the prevalence of CVD increases 4.56 times for every unit increase in MLR in people over 60. In the 20–60 age range, however, no discernible correlation is found. And we discovered that age, sex, BMI, diabetes, and hypertension had no substantial impact on the relationship between MLR, SIRI, and CAR with CVD. Our findings suggest that MLR could serve as a cost-effective tool for risk stratification in primary care settings. Patients with elevated MLR may benefit from intensified cardiovascular monitoring or early preventive interventions, such as anti-inflammatory therapies or lifestyle modifications.

In our study, we also delved into the relationship between CVD and various inflammatory indicators. The SIRI has drawn more attention recently due to its capacity to predict the risk of CVD. Prior research has mostly concentrated on the strong correlation between SIRI and the death rate from CVD [47–50]. According to our research, the prevalence of CVD rises by 14% for every unit increase in SIRI. SIRI functions as an inflammation marker that combines three immunological pathways: lymphocytes, which represent immune control, and neutrophils and monocytes, which explain chronic inflammatory responses. Such a combination may offer a comprehensive evaluation of immunological balance and systemic inflammation [51, 52]. Furthermore, We observed a non-linear relationship and a positive association between CHF and CAR, which aligns with findings from other studies [53, 54]. Inflammatory states are linked to CRP, which may indicate the severity of CHF or be brought on by a number of other reasons. Its expression rises in inflammatory situations because it is an acute-phase inflammatory protein. Another liver-produced protein, albumin, serves as a general indicator of nutritional status, overall health, and response to treatment. In CHF patients, hypoalbuminemia may be a sign of nutritional deficiency, which frequently results in worse outcomes [54].

Inflammation plays a crucial role in CVD, primarily through mechanisms such as endothelial dysfunction, atherosclerosis, platelet activation, and oxidative stress. Firstly, the inflammatory response facilitates the accumulation of macrophages and lipids within the arterial intima, leading to the formation of foam cells and exacerbating plaque development, ultimately resulting in vascular lumen narrowing [55]. Secondly, inflammatory mediators can activate platelets, promoting thrombosis and increasing the risk of myocardial infarction and other cardiovascular events [56]. Furthermore, oxidative stress induced by chronic inflammation can damage endothelial cells, further promoting CVD progression [11].

The present study has several strengths, including a large, nationally representative sample and rigorous adjustment for confounding variables. However, as a cross-sectional study, it cannot establish causality. Additionally, CVD events were self-reported, which may introduce recall bias. Despite adjusting for a comprehensive set of covariates, unmeasured confounders such as specific comorbidities may influence the observed associations. Finally, our findings may not be generalizable to other populations or ethnic groups. Future studies should validate these findings in external or non-American populations to enhance generalizability and explore potential ethnic or regional variations. Longitudinal studies and more sophisticated modeling approaches, such as causal inference methods, are needed to establish causality and further elucidate the role of MLR in CVD.

## Conclusion

Compared with other inflammatory indicators (SIRI, AISI, and CAR), MLR appears to be a better inflammatory index for predicting CVD, CHF, CHD, angina pectoris, heart attack, and stroke. American adults with elevated MLR and SIRI should be aware of the possible harm caused by CVD. Causal inference is, however, limited by the cross-sectional design and dependence on self-reported data. Further longitudinal studies are needed to validate these findings.

## Electronic supplementary material

Below is the link to the electronic supplementary material.

Supplementary Material 1

## Data Availability

All data regarding this study are available on the NHANES website (https://www.cdc.gov/nchs/nhanes/).

## Abbreviations

MLR: Monocyte-to-lymphocyte ratio
CVD: Cardiovascular diseases
NHANES: National Health and Nutrition Examination Survey
CHF: Congestive heart failure
CHD: Coronary heart disease
SIRI: System inflammation response index
AISI: Aggregate index of systemic inflammation
CAR: C-reactive protein-to-albumin ratio
ROC: Receiver operating characteristic
MC: Monocyte count
LC: Lymphocyte count
T2D: With type 2 diabetes
NCHS: National Center for Health Statistics
CBC: Complete blood count
ALB: Albumin
BCP: Bromocresol purple
CRP: C-reactive protein
PC: Platelet count
NC: Neutrophil count
PIR: Family income to poverty ratio
BMI: Body mass index
TC: Total cholesterol
HDL-C: High-density lipoprotein-cholesterol
AST: Aspartate aminotransferase
ALT: Alanine aminotransferase
DKD: Diabetic kidney disease
COPD: Chronic obstructive pulmonary disease
RA: Rheumatoid arthritis
SBP: Systolic blood pressure
DBP: Diastolic blood pressure
FPG: Fasting plasma glucose
HbA1c: Hemoglobin A1c
eGFR: Estimated glomerular filtration rate
UACR: Urinary albumin-to-creatinine ratio
CDC: Centers for Disease Control
SE: Standard error
GAM: Generalized additive models
AUC: Area under the curve
SDS: Standardized Domination Statistic
HD: Hemodialysis
PD: Peritoneal dialysis
CKD: Chronic kidney disease
NO: Nitric oxide
