# Supplementary material for: Association between monocyte-to-lymphocyte ratio and cardiovascular diseases: insights from NHANES data
Source: Diabetol Metab Syndr. 2025 Mar 24;17:98. doi: 10.1186/s13098-025-01640-9 (PMC11931847; doi:10.1186/s13098-025-01640-9)
Supplement: Supplementary file 1 — Supplementary Material 1 [file 13098_2025_1640_MOESM1_ESM.docx]

**[Supplementary Table S1](https://www.ncbi.nlm.nih.gov/pmc/articles/PMC10494374/bin/12882_2023_3316_MOESM2_ESM.tif)** | Association between MLR and other inflammatory biomarkers with CHF, CHD, angina pectoris, heart attack, and stroke.

| **Index** | **Outcome** | **Continuous or categories** | Model 1^3^ | | Model 2^4^ | | [Model 3](https://www.ncbi.nlm.nih.gov/pmc/articles/PMC8987107/table/T2/?report=objectonly#t2fna)^5^ | |
| --- | --- | --- | --- | --- | --- | --- | --- | --- |
|  |  |  | OR^1^ (95%CI^2^) | *P-* value | OR (95%CI) | *P-* value | OR (95%CI) | *P-* value |
| ****MLR**** | ****CHF**** | **MLR** as continuous variable | 34.35 (25.58, 46.13) | <0.0001 | 8.89 (6.46, 12.25) | <0.0001 | 6.71 (2.15, 21.00) | 0.0011 |
|  |  | Tertile 1 | Reference |  | Reference |  | Reference |  |
|  |  | Tertile 2 | 1.20 (1.01, 1.42) | 0.0347 | 1.03 (0.86, 1.22) | 0.7630 | 0.90 (0.52, 1.54) | 0.6966 |
|  |  | Tertile 3 | 3.35 (2.90, 3.87) | <0.0001 | 1.97 (1.69, 2.30) | <0.0001 | 1.66 (1.03, 2.70) | 0.0386 |
|  |  | *P* for trend | <0.0001 |  | <0.0001 |  | 0.0069 |  |
|  | ****CHD**** | **MLR** as continuous variable | 27.35 (20.71, 36.11) | <0.0001 | 3.11 (2.26, 4.29) | <0.0001 | 3.12 (1.18, 8.24) | 0.0219 |
|  |  | Tertile 1 | Reference |  | Reference |  | Reference |  |
|  |  | Tertile 2 | 1.46 (1.26, 1.70) | <0.0001 | 1.04 (0.89, 1.21) | 0.6394 | 1.43 (0.88, 2.33) | 0.1485 |
|  |  | Tertile 3 | 3.49 (3.05, 3.98) | <0.0001 | 1.43 (1.24, 1.66) | <0.0001 | 1.74 (1.10, 2.76) | 0.0182 |
|  |  | *P* for trend | <0.0001 |  | <0.0001 |  | 0.0199 |  |
|  | **Angina pectoris** | **MLR** as continuous variable | 10.36 (7.47, 14.36) | <0.0001 | 1.83 (1.23, 2.72) | 0.0029 | 0.99 (0.31, 3.15) | 0.9905 |
|  |  | Tertile 1 | Reference |  | Reference |  | Reference |  |
|  |  | Tertile 2 | 1.32 (1.12, 1.55) | 0.0009 | 1.03 (0.87, 1.22) | 0.6936 | 1.16 (0.70, 1.92) | 0.5540 |
|  |  | Tertile 3 | 2.30 (1.98, 2.67) | <0.0001 | 1.16 (0.99, 1.37) | 0.0682 | 1.17 (0.72, 1.90) | 0.5196 |
|  |  | *P* for trend | <0.0001 |  | 0.0408 |  | 0.5948 |  |
|  | **Heart attack** | **MLR** as continuous variable | 22.14 (16.83, 29.12) | <0.0001 | 3.23 (2.37, 4.41) | <0.0001 | 2.45 (0.92, 6.53) | 0.0722 |
|  |  | Tertile 1 | Reference |  | Reference |  | Reference |  |
|  |  | Tertile 2 | 1.28 (1.11, 1.48) | 0.0006 | 0.98 (0.84, 1.13) | 0.7431 | 1.17 (0.76, 1.81) | 0.4793 |
|  |  | Tertile 3 | 3.01 (2.66, 3.41) | <0.0001 | 1.42 (1.24, 1.62) | <0.0001 | 1.40 (0.93, 2.12) | 0.1114 |
|  |  | *P* for trend | <0.0001 |  | <0.0001 |  | 0.0940 |  |
|  | **Stroke** | **MLR** as continuous variable | 8.56 (6.35, 11.54) | <0.0001 | 2.07 (1.46, 2.94) | <0.0001 | 3.39 (1.23, 9.35) | 0.0184 |
|  |  | Tertile 1 | Reference |  | Reference |  | Reference |  |
|  |  | Tertile 2 | 1.02 (0.89, 1.17) | 0.7615 | 0.89 (0.78, 1.03) | 0.1191 | 0.82 (0.52, 1.27) | 0.3627 |
|  |  | Tertile 3 | 1.81 (1.60, 2.05) | <0.0001 | 1.10 (0.96, 1.25) | 0.1762 | 0.99 (0.65, 1.50) | 0.9696 |
|  |  | *P* for trend | <0.0001 |  | 0.0382 |  | 0.7727 |  |
| ****SIRI**** | ****CHF**** | SIRI as continuous variable | 1.44 (1.39, 1.50) | <0.0001 | 1.32 (1.26, 1.37) | <0.0001 | 1.10 (0.97, 1.25) | 0.1308 |
|  |  | Tertile 1 | Reference |  | Reference |  | Reference |  |
|  |  | Tertile 2 | 1.31 (1.11, 1.55) | 0.0014 | 1.27 (1.07, 1.51) | 0.0055 | 0.87 (0.51, 1.49) | 0.6149 |
|  |  | Tertile 3 | 3.34 (2.90, 3.86) | <0.0001 | 2.62 (2.24, 3.05) | <0.0001 | 1.48 (0.91, 2.40) | 0.1123 |
|  |  | *P* for trend | <0.0001 |  | <0.0001 |  | 0.0245 |  |
|  | ****CHD**** | SIRI as continuous variable | 1.39 (1.34, 1.44) | <0.0001 | 1.15 (1.11, 1.20) | <0.0001 | 1.08 (0.96, 1.21) | 0.2033 |
|  |  | Tertile 1 | Reference |  | Reference |  | Reference |  |
|  |  | Tertile 2 | 1.60 (1.39, 1.85) | <0.0001 | 1.22 (1.05, 1.42) | 0.0088 | 0.93 (0.60, 1.46) | 0.7662 |
|  |  | Tertile 3 | 3.11 (2.73, 3.54) | <0.0001 | 1.63 (1.42, 1.88) | <0.0001 | 1.25 (0.82, 1.89) | 0.2989 |
|  |  | *P* for trend | <0.0001 |  | <0.0001 |  | 0.1292 |  |
|  | **Angina pectoris** | SIRI as continuous variable | 1.29 (1.24, 1.35) | <0.0001 | 1.13 (1.08, 1.18) | <0.0001 | 1.00 (0.89, 1.13) | 0.9916 |
|  |  | Tertile 1 | Reference |  | Reference |  | Reference |  |
|  |  | Tertile 2 | 1.53 (1.30, 1.80) | <0.0001 | 1.25 (1.05, 1.47) | 0.0101 | 1.24 (0.77, 2.01) | 0.3761 |
|  |  | Tertile 3 | 2.40 (2.06, 2.79) | <0.0001 | 1.47 (1.25, 1.73) | <0.0001 | 0.92 (0.56, 1.49) | 0.7331 |
|  |  | *P* for trend | <0.0001 |  | <0.0001 |  | 0.3895 |  |
|  | **Heart attack** | SIRI as continuous variable | 1.39 (1.34, 1.44) | <0.0001 | 1.20 (1.16, 1.25) | <0.0001 | 1.08 (0.97, 1.21) | 0.1520 |
|  |  | Tertile 1 | Reference |  | Reference |  | Reference |  |
|  |  | Tertile 2 | 1.46 (1.28, 1.68) | <0.0001 | 1.24 (1.07, 1.43) | 0.0035 | 0.81 (0.53, 1.23) | 0.3201 |
|  |  | Tertile 3 | 2.88 (2.54, 3.26) | <0.0001 | 1.79 (1.56, 2.04) | <0.0001 | 1.21 (0.82, 1.78) | 0.3470 |
|  |  | *P* for trend | <0.0001 |  | <0.0001 |  | 0.0949 |  |
|  | **Stroke** | SIRI as continuous variable | 1.26 (1.22, 1.31) | <0.0001 | 1.16 (1.11, 1.21) | <0.0001 | 1.10 (0.98, 1.23) | 0.0956 |
|  |  | Tertile 1 | Reference |  | Reference |  | Reference |  |
|  |  | Tertile 2 | 1.13 (0.99, 1.30) | 0.0700 | 1.13 (0.98, 1.30) | 0.0956 | 1.02 (0.65, 1.60) | 0.9305 |
|  |  | Tertile 3 | 1.98 (1.75, 2.24) | <0.0001 | 1.60 (1.40, 1.83) | <0.0001 | 1.30 (0.85, 2.00) | 0.2233 |
|  |  | *P* for trend | <0.0001 |  | <0.0001 |  | 0.1464 |  |
| **AISI** | ****CHF**** | AISI as continuous variable | 1.01 (1.00, 1.01) | <0.0001 | 1.01 (1.01, 1.02) | <0.0001 | 1.01 (0.99, 1.02) | 0.4966 |
|  |  | Tertile 1 | Reference |  | Reference |  | Reference |  |
|  |  | Tertile 2 | 1.20 (1.03, 1.39) | <0.0001 | 1.20 (1.03, 1.40) | 0.0175 | 0.83 (0.53, 1.32) | 0.4360 |
|  |  | Tertile 3 | 2.04 (1.78, 2.33) | <0.0001 | 1.87 (1.62, 2.16) | <0.0001 | 0.99 (0.64, 1.53) | 0.9751 |
|  |  | *P* for trend | <0.0001 |  | <0.0001 |  | 0.8224 |  |
|  | ****CHD**** | AISI as continuous variable | 1.01 (1.00, 1.01) | <0.0001 | 1.01 (1.01, 1.02) | 0.0375 | 1.00 (0.99, 1.01) | 0.6327 |
|  |  | Tertile 1 | Reference |  | Reference |  | Reference |  |
|  |  | Tertile 2 | 1.32 (1.16, 1.50) | <0.0001 | 1.12 (0.98, 1.28) | 0.1065 | 0.96 (0.66, 1.41) | 0.8371 |
|  |  | Tertile 3 | 1.78 (1.58, 2.01) | <0.0001 | 1.25 (1.10, 1.43) | 0.0006 | 0.91 (0.63, 1.31) | 0.6142 |
|  |  | *P* for trend | <0.0001 |  | 0.0006 |  | 0.6093 |  |
|  | **Angina pectoris** | AISI as continuous variable | 1.01 (1.01, 1.02) | <0.0001 | 1.01 (1.01, 1.02) | 0.0069 | 1.00 (0.99, 1.01) | 0.4304 |
|  |  | Tertile 1 | Reference |  | Reference |  | Reference |  |
|  |  | Tertile 2 | 1.26 (1.08, 1.47) | 0.0031 | 1.11 (0.95, 1.30) | 0.2056 | 0.97 (0.63, 1.49) | 0.8861 |
|  |  | Tertile 3 | 1.76 (1.53, 2.04) | <0.0001 | 1.34 (1.15, 1.56) | <0.0001 | 0.79 (0.51, 1.22) | 0.2845 |
|  |  | *P* for trend | <0.0001 |  | <0.0001 |  | 0.2439 |  |
|  | **Heart attack** | AISI as continuous variable | 1.01 (1.01, 1.02) | <0.0001 | 1.01 (1.00, 1.01) | <0.0001 | 1.01 (0.99, 1.01) | 0.3802 |
|  |  | Tertile 1 | Reference |  | Reference |  | Reference |  |
|  |  | Tertile 2 | 1.22 (1.08, 1.38) | 0.0020 | 1.12 (0.99, 1.28) | 0.0804 | 0.89 (0.61, 1.29) | 0.5270 |
|  |  | Tertile 3 | 1.85 (1.65, 2.08) | <0.0001 | 1.49 (1.31, 1.68) | <0.0001 | 0.99 (0.70, 1.42) | 0.9761 |
|  |  | *P* for trend | <0.0001 |  | <0.0001 |  | 0.8714 |  |
|  | **Stroke** | AISI as continuous variable | 1.01 (1.01, 1.02) | <0.0001 | 1.01 (1.00, 1.01) | <0.0001 | 1.01 (0.99, 1.02) | 0.1929 |
|  |  | Tertile 1 | Reference |  | Reference |  | Reference |  |
|  |  | Tertile 2 | 1.05 (0.92, 1.20) | 0.4598 | 1.08 (0.94, 1.23) | 0.2811 | 1.10 (0.73, 1.65) | 0.6433 |
|  |  | Tertile 3 | 1.53 (1.36, 1.73) | <0.0001 | 1.45 (1.28, 1.65) | <0.0001 | 1.12 (0.75, 1.67) | 0.5726 |
|  |  | *P* for trend | <0.0001 |  | <0.0001 |  | 0.6149 |  |
| **CAR** | ****CHF**** | CAR as continuous variable | 2.10 (1.79, 2.47) | <0.0001 | 1.87 (1.57, 2.23) | <0.0001 | 1.81 (1.20, 2.72) | 0.0045 |
|  |  | Tertile 1 | Reference |  | Reference |  | Reference |  |
|  |  | Tertile 2 | 1.79 (1.43, 2.23) | <0.0001 | 1.36 (1.09, 1.71) | 0.0077 | 1.49 (1.01, 2.19) | 0.0458 |
|  |  | Tertile 3 | 3.05 (2.48, 3.76) | <0.0001 | 2.34 (1.89, 2.90) | <0.0001 | 1.50 (1.01, 2.23) | 0.0420 |
|  |  | *P* for trend | <0.0001 |  | <0.0001 |  | 0.1657 |  |
|  | ****CHD**** | CAR as continuous variable | 1.39 (1.16, 1.68) | .0004 | 1.22 (0.99, 1.49) | 0.0547 | 0.85 (0.46, 1.58) | 0.6152 |
|  |  | Tertile 1 | Reference |  | Reference |  | Reference |  |
|  |  | Tertile 2 | 1.53 (1.29, 1.81) | <0.0001 | 1.18 (0.99, 1.41) | 0.0591 | 1.04 (0.77, 1.40) | 0.8135 |
|  |  | Tertile 3 | 1.65 (1.40, 1.95) | <0.0001 | 1.38 (1.16, 1.65) | 0.0003 | 0.84 (0.61, 1.16) | 0.2996 |
|  |  | *P* for trend | <0.0001 |  | 0.0006 |  | 0.1724 |  |
|  | **Angina pectoris** | CAR as continuous variable | 1.67 (1.41, 1.99) | <0.0001 | 1.49 (1.23, 1.79) | <0.0001 | 1.82 (1.20, 2.74) | 0.0045 |
|  |  | Tertile 1 | Reference |  | Reference |  | Reference |  |
|  |  | Tertile 2 | 1.45 (1.18, 1.77) | 0.0004 | 1.13 (0.92, 1.39) | 0.2511 | 0.87 (0.60, 1.28) | 0.4939 |
|  |  | Tertile 3 | 2.01 (1.66, 2.43) | <0.0001 | 1.62 (1.33, 1.98) | <0.0001 | 1.11 (0.76, 1.62) | 0.6054 |
|  |  | *P* for trend | <0.0001 |  | <0.0001 |  | 0.3049 |  |
|  | **Heart attack** | CAR as continuous variable | 1.70 (1.46, 1.99) | <0.0001 | 1.52 (1.28, 1.80) | <0.0001 | 1.33 (0.87, 2.03) | 0.1900 |
|  |  | Tertile 1 | Reference |  | Reference |  | Reference |  |
|  |  | Tertile 2 | 1.46 (1.23, 1.73) | <0.0001 | 1.14 (0.96, 1.36) | 0.1318 | 1.10 (0.81, 1.49) | 0.5402 |
|  |  | Tertile 3 | 1.92 (1.63, 2.25) | <0.0001 | 1.61 (1.36, 1.91) | <0.0001 | 1.14 (0.84, 1.56) | 0.3951 |
|  |  | *P* for trend | <0.0001 |  | <0.0001 |  | 0.4713 |  |
|  | **Stroke** | CAR as continuous variable | 1.54 (1.29, 1.83) | <0.0001 | 1.28 (1.05, 1.55) | 0.0147 | 1.23 (0.77, 1.96) | 0.3821 |
|  |  | Tertile 1 | Reference |  | Reference |  | Reference |  |
|  |  | Tertile 2 | 1.55 (1.27, 1.88) | <0.0001 | 1.19 (0.97, 1.45) | 0.0959 | 1.14 (0.80, 1.61) | 0.4707 |
|  |  | Tertile 3 | 2.24 (1.86, 2.69) | <0.0001 | 1.64 (1.35, 1.98) | <0.0001 | 1.36 (0.96, 1.93) | 0.0830 |
|  |  | *P* for trend | <0.0001 |  | <0.0001 |  | 0.0740 |  |

In sensitivity analysis, MLR, SIRI, AISI, and CAR were converted from continuous variables to categorical variables (tertiles).

^1^OR: Odd ratio.

^2^95% CI: 95% confidence interval.

^3^Model 1: No covariates were adjusted.

^4^Model 2: Adjusted for age, sex, and race.

^5^Model 3: Adjusted for sex, age, race, marital status, education level, BMI, smoking status, alcohol consumption, TC, HDL-C, AST, ALT, PIR, total bilirubin, serum total calcium, RA, DKD, asthma, close relative had heart attack, COPD, hypertension and diabetes.

**[Supplementary Table S2](https://www.ncbi.nlm.nih.gov/pmc/articles/PMC10494374/bin/12882_2023_3316_MOESM2_ESM.tif)** | Threshold effect analysis of MLR and other inflammatory biomarkers on CHF, CHD, angina pectoris, heart attack, and stroke using a two-piecewise linear regression model in Model 3.

|  | MLR | SIRI | AISI | CAR |
| --- | --- | --- | --- | --- |
|  | OR^1^ (95%CI^2^) *P-* value | OR^1^ (95%CI^2^) *P-* value | OR^1^ (95%CI^2^) *P-* value | OR^1^ (95%CI^2^) *P-* value |
| **CHF** |  |  |  |  |
| **Fitting by standard linear model** | 6.71 (2.15, 21.00) 0.0011 | 1.10 (0.97, 1.25) 0.1308 | 1.01 (0.99, 1.02)0.4966 | 1.81 (1.20, 2.72) 0.0045 |
| **Fitting by two-piecewise linear model** |  |  |  |  |
| Breakpoint (K) | 0.48 | 2.83 | 112.65 | 0.02 |
| OR1(< K ) | 22.85 (3.39, 53.83) 0.0013 | 1.38 (1.07, 1.77) 0.0119 | 0.99 (0.98, 1.01) 0.1825 | 3.22 (2.82, 5.68) 0.0122 |
| OR2(> K ) | 1.78 (0.26, 12.30) 0.5611 | 0.98 (0.85, 1.14) 0.8328 | 1.00 (0.99, 1.01) 0.3727 | 1.67 (1.09, 2.54) 0.0172 |
| OR2 / OR1 | 0.08 (0.01, 1.73) 0.1068 | 0.71 (0.52, 0.99) 0.0403 | 1.01 (0.99, 1.02) 0.1754 | 0.02 (0.01, 0.03) 0.0131 |
| Logarithmic likelihood ratio test P-value | 0.116 | 0.045 | 0.193 | 0.007 |
| ****CHD**** |  |  |  |  |
| **Fitting by standard linear model** | 3.12 (1.18, 8.24) 0.0219 | 1.08 (0.96, 1.21) 0.2033 | 1.00 (0.99, 1.01) 0.6327 | 0.85 (0.46, 1.58) 0.6152 |
| **Fitting by two-piecewise linear model** |  |  |  |  |
| Breakpoint (K) | 0.23 | 1.49 | 556.16 | 0.01 |
| OR1(< K ) | 7.03 (0.03, 18.46) 0.2918 | 1.59 (0.99, 2.54) 0.0545 | 0.99 (0.98, 1.01) 0.3489 | 0.59 (0.01, 1.55) 0.0980 |
| OR2(> K ) | 2.64 (0.92, 7.60) 0.0709 | 1.01 (0.89, 1.15) 0.8340 | 1.00 (0.99, 1.01) 0.8573 | 0.81 (0.43, 1.53) 0.5118 |
| OR2 / OR1 | 0.04 (0.01, 41.89) 0.4353 | 0.64 (0.38, 1.08) 0.0938 | 1.00 (0.99, 1.01) 0.4179 | 0.68 (0.01, 6.52) 0.0974 |
| Logarithmic likelihood ratio test P-value | 0.425 | 0.094 | 0.414 | 0.076 |
| **Angina pectoris** |  |  |  |  |
| **Fitting by standard linear model** | 0.99 (0.31, 3.15) 0.9905 | 1.00 (0.89, 1.13) 0.9916 | 1.00 (0.99, 1.01) 0.4304 | 1.82 (1.20, 2.74) 0.0045 |
| **Fitting by two-piecewise linear model** |  |  |  |  |
| Breakpoint (K) | 0.18 | 0.4 | 516.6 | 0.02 |
| OR1(< K ) | 0.02 (0.01, 11.24) 0.1861 | 3.21 (0.01, 5.14) 0.2268 | 1.00 (0.99, 1.01)0.2489 | 0.02 (0.01, 1.58) 0.4228 |
| OR2(> K ) | 1.19 (0.37, 3.89) 0.7678 | 0.99 (0.88, 1.12) 0.8768 | 0.99 (0.98, 1.01) 0.9405 | 1.86 (1.23, 2.81) 0.0031 |
| OR2 / OR1 | 3.72 (0.01, 13.74) 0.1863 | 0.02 (0.01, 3.79) 0.2266 | 0.99 (0.98, 1.01) 0.3548 | 6.40 (0.01, 9.58) 0.4044 |
| Logarithmic likelihood ratio test P-value | 0.213 | 0.129 | 0.356 | 0.412 |
| **Heart attack** |  |  |  |  |
| **Fitting by standard linear model** | 2.45 (0.92, 6.53) 0.0722 | 1.08 (0.97, 1.21) 0.1520 | 1.01 (0.99, 1.02) 0.3802 | 1.33 (0.87, 2.03) 0.1900 |
| **Fitting by two-piecewise linear model** |  |  |  |  |
| Breakpoint (K) | 0.23 | 2.83 | 155.62 | 0.37 |
| OR1(< K ) | 29.80 (0.03, 35.13) 0.3470 | 1.23 (0.99, 1.52) 0.0519 | 1.01 (0.99, 1.01) 0.1673 | 3.23 (1.03, 10.19) 0.0452 |
| OR2(> K ) | 2.10 (0.72, 6.12) 0.1724 | 1.00 (0.86, 1.16) 0.9977 | 1.00 (0.99, 1.01) 0.2212 | 0.94 (0.49, 1.82) 0.8530 |
| OR2 / OR1 | 0.07 (0.01, 17.93) 0.4838 | 0.81 (0.61, 1.08) 0.1559 | 1.00 (0.99, 1.01) 0.1500 | 0.29 (0.06, 1.32) 0.1091 |
| Logarithmic likelihood ratio test P-value | 0.477 | 0.166 | 0.159 | 0.106 |
| **Stroke** |  |  |  |  |
| **Fitting by standard linear model** | 3.39 (1.23, 9.35) 0.0184 | 1.10 (0.98, 1.23) 0.0956 | 1.01 (0.99, 1.02) 0.1929 | 1.23 (0.77, 1.96) 0.3821 |
| **Fitting by two-piecewise linear model** |  |  |  |  |
| Breakpoint (K) | 0.44 | 0.4 | 250 | 0.11 |
| OR1(< K ) | 1.43 (0.21, 9.75) 0.7121 | 12.06 (0.01, 26.58) 0.3048 | 1.01 (0.99, 1.02) 0.3008 | 8.42 (0.22, 16.38) 0.2496 |
| OR2(> K ) | 6.62 (1.29, 34.10) 0.0238 | 1.09 (0.98, 1.22) 0.1208 | 1.00 (0.99, 1.01) 0.3886 | 1.09 (0.63, 1.89) 0.7623 |
| OR2 / OR1 | 4.62 (0.25, 85.15) 0.3036 | 0.02 (0.01, 90.02) 0.3143 | 1.01 (0.99, 1.02) 0.3862 | 0.13 (0.01, 5.97) 0.2955 |
| Logarithmic likelihood ratio test P-value | 0.299 | 0.261 | 0.382 | 0.295 |

Adjusted for sex, age, race, marital status, education level, BMI, smoking status, alcohol consumption, TC, HDL-C, AST, ALT, PIR, total bilirubin, serum total calcium, RA, DKD, asthma, close relative had heart attack, COPD, hypertension and diabetes.

^1^OR: Odd ratio.

^2^95% CI: 95% confidence interval.

**[Supplementary Table S3](https://www.ncbi.nlm.nih.gov/pmc/articles/PMC10494374/bin/12882_2023_3316_MOESM2_ESM.tif)** | Interaction effects of continuous variables (BMI and SBP) with MLR, SIRI, AISI, and CAR inpredicting CVD, CHF, CHD, angina Pectoris, heart Attack, and stroke.

| **Outcome** | **MLR × BMI** | **MLR × SBP** | **SIRI × BMI** | **SIRI × SBP** | **AISI × BMI** | **AISI × SBP** | **CAR × BMI** | **CAR × SBP** |
| --- | --- | --- | --- | --- | --- | --- | --- | --- |
|  | **OR^1^ (95%CI^2^) *P*- value** | | | | | | | |
| **CVD** | 1.06 (1.03,1.08) <0.0001 | 1.01 (1.00,1.01) 0.0059 | 1.00 (1.00,1.01) 0.0040 | 1.00 (0.99,1.01) 0.0520 | 1.00 (0.99,1.01) 0.2027 | 1.00 (0.99,1.01) 0.5786 | 1.00 (0.99,1.02) 0.4312 | 1.00 (0.99,1.01) 0.7971 |
| **CHF** | 1.09 (1.05,1.13) <0.0001 | 1.01 (1.00,1.02) 0.0191 | 1.01 (1.00,1.01) 0.0062 | 1.00 (0.99,1.01) 0.3188 | 1.00 (0.99,1.01) 0.1035 | 1.00 (0.99,1.01) 0.8440 | 1.01 (0.99,1.03) 0.0898 | 1.00 (0.99,1.01) 0.7926 |
| **CHD** | 1.05 (1.02,1.08) 0.0030 | 1.01 (0.99,1.01) 0.0884 | 1.00 (0.99,1.01) 0.1073 | 1.00 (0.99,1.00) 0.6418 | 1.00 (0.99,1.01) 0.7723 | 1.00 (0.99,1.01) 0.2543 | 0.99 (0.97,1.01) 0.4077 | 1.00 (0.99,1.01) 0.6913 |
| **Angina Pectoris** | 1.02 (0.98,1.05) 0.3749 | 0.99 (0.99,1.01) 0.8173 | 1.00 (0.99,1.01) 0.5043 | 1.00 (0.99,1.00) 0.7866 | 1.00 (0.99,1.01) 0.6636 | 1.00 (0.99,1.01) 0.2416 | 1.01 (0.99,1.02) 0.4844 | 1.00 (0.99,1.01) 0.2732 |
| **Heart Attack** | 1.04 (1.01,1.08) 0.0043 | 1.01 (1.00,1.02) 0.0457 | 1.00 (0.99,1.01) 0.0942 | 1.00 (0.99,1.01) 0.4376 | 1.00 (0.99,1.01) 0.3903 | 1.00 (0.99,1.01) 0.9862 | 1.01 (0.99,1.02) 0.2695 | 1.00 (0.99,1.01) 0.2029 |
| **Stroke** | 1.02 (0.99,1.06) 0.1699 | 1.01 (1.00,1.02) 0.0272 | 1.00 (0.99,1.01) 0.5642 | 1.00 (0.99,1.01) 0.0558 | 1.00 (0.99,1.01) 0.8833 | 1.00 (0.99,1.01) 0.0874 | 1.01 (0.99,1.03) 0.4101 | 1.00 (0.99,1.01) 0.7906 |

^1^OR: Odd ratio.

^2^95% CI: 95% confidence interval.

**[Supplementary Table S4](https://www.ncbi.nlm.nih.gov/pmc/articles/PMC10494374/bin/12882_2023_3316_MOESM2_ESM.tif) |** Comparison of AUC values between MLR and other inflammatory biomarkers.

| Test | AUC^1^ | 95%CI ^2^ low | 95%CI upp | Best threshold | Specificity | Sensitivity | *P* for different in AUC |
| --- | --- | --- | --- | --- | --- | --- | --- |
| **CVD** |  |  |  |  |  |  |  |
| MLR | 0.6450 | 0.6354 | 0.6547 | 0.2968 | 0.6861 | 0.5403 | Reference |
| SIRI | 0.6298 | 0.6201 | 0.6394 | 1.2130 | 0.6460 | 0.5519 | <0.0001 |
| AISI | 0.5715 | 0.5616 | 0.5814 | 295.2300 | 0.6196 | 0.4943 | <0.0001 |
| CAR | 0.5938 | 0.5813 | 0.6062 | 0.0342 | 0.4124 | 0.7172 | <0.0001 |
| **CHF** |  |  |  |  |  |  |  |
| MLR | 0.6731 | 0.6577 | 0.6884 | 0.3066 | 0.6993 | 0.5726 | Reference |
| SIRI | 0.6601 | 0.6448 | 0.6755 | 1.2897 | 0.6802 | 0.5805 | <0.0001 |
| AISI | 0.5946 | 0.5785 | 0.6106 | 294.5125 | 0.6137 | 0.5451 | <0.0001 |
| CAR | 0.6422 | 0.6221 | 0.6623 | 0.0641 | 0.5963 | 0.6048 | <0.0001 |
| **CHD** |  |  |  |  |  |  |  |
| MLR | 0.6638 | 0.6502 | 0.6774 | 0.3037 | 0.6941 | 0.5587 | Reference |
| SIRI | 0.6453 | 0.6319 | 0.6587 | 1.0689 | 0.5514 | 0.6754 | <0.0001 |
| AISI | 0.5694 | 0.5556 | 0.5833 | 294.9971 | 0.6143 | 0.4971 | <0.0001 |
| CAR | 0.5630 | 0.5454 | 0.5805 | 0.0312 | 0.3790 | 0.7215 | <0.0001 |
| **Angina pectoris** |  |  |  |  |  |  |  |
| MLR | 0.6106 | 0.5936 | 0.6275 | 0.3150 | 0.7242 | 0.4429 | Reference |
| SIRI | 0.6105 | 0.5940 | 0.6271 | 1.1544 | 0.6000 | 0.5723 | <0.0001 |
| AISI | 0.5655 | 0.5485 | 0.5824 | 324.4650 | 0.6678 | 0.4403 | <0.0001 |
| CAR | 0.5900 | 0.5692 | 0.6107 | 0.0893 | 0.6916 | 0.4338 | <0.0001 |
| **Heart attack** |  |  |  |  |  |  |  |
| MLR | 0.6492 | 0.6358 | 0.6627 | 0.2793 | 0.6154 | 0.6215 | Reference |
| SIRI | 0.6383 | 0.6250 | 0.6516 | 1.1583 | 0.6068 | 0.6045 | <0.0001 |
| AISI | 0.5783 | 0.5645 | 0.5922 | 298.4493 | 0.6216 | 0.5074 | <0.0001 |
| CAR | 0.5900 | 0.5725 | 0.6075 | 0.0523 | 0.5326 | 0.5994 | <0.0001 |
| **Stroke** |  |  |  |  |  |  |  |
| MLR | 0.6298 | 0.6210 | 0.6386 | 0.2968 | 0.6885 | 0.5130 | Reference |
| SIRI | 0.6187 | 0.6100 | 0.6274 | 1.2107 | 0.6484 | 0.5362 | <0.0001 |
| AISI | 0.5676 | 0.5587 | 0.5764 | 2.0909 | 0.6529 | 0.4553 | <0.0001 |
| CAR | 0.5957 | 0.5844 | 0.6069 | 0.0293 | 0.3722 | 0.7569 | <0.0001 |

^1^ AUC: area under the curve.

^2^ 95% CI: 95% confidence interval.

**[Supplementary Table S5](https://www.ncbi.nlm.nih.gov/pmc/articles/PMC10494374/bin/12882_2023_3316_MOESM2_ESM.tif)** | Calculation of the Relative Importance of MLR, SIRI, AISI, CAR in predicting CVD, CHF, CHD, Angina pectoris, Heart attack, and Stroke using Standardized Domination Statistic.

| Outcome (Odds Ratio of Importance) | MLR | SIRI | AISI | CAR |
| --- | --- | --- | --- | --- |
| **CVD** | 0.3430 | 0.3379 | 0.2096 | 0.1095 |
| **CHF** | 0.3354 | 0.3301 | 0.2080 | 0.1265 |
| **CHD** | 0.3429 | 0.3420 | 0.2258 | 0.0893 |
| **Angina pectoris** | 0.3134 | 0.2866 | 0.1913 | 0.2087 |
| **Heart attack** | 0.3448 | 0.3392 | 0.2046 | 0.1114 |
| **Stroke** | 0.3680 | 0.3504 | 0.2071 | 0.0745 |

Odds Ratio of Importance indicates the relative importance of each marker in predicting the outcome.
